# Evading regulation: young people’s exposure to harmful commodity marketing in the social media feeds of their favourite influencers

**DOI:** 10.1093/heapro/daag037

**Published:** 2026-03-14

**Authors:** Antonia C Lyons, Ian Goodwin, Jessica Young, Cassandra Burton-Wood

**Affiliations:** Centre for Addiction Research, Social and Community Health, University of Auckland, Private Bag 92019, Auckland 1142, New Zealand; School of Social Sciences, University of Auckland, Private Bag 92019, Auckland 1142, New Zealand; School of Health, Victoria University of Wellington, PO Box 600, Wellington 6140, New Zealand; Laidlaw College, 70 Condell Avenue, Papanui, Christchurch 8053, New Zealand

**Keywords:** social media, influencers, marketing, harmful commodities, vape, alcohol, tobacco

## Abstract

Increasingly companies use social media influencers to market unhealthy products to young people, although we know little about the influencers young people follow and the content influencers post. This study aimed to identify influencers young people follow, examine their exposure to harmful commodity content in influencer accounts, and examine the Instagram accounts of the most popular influencers for harmful commodity content and marketing. In Stage 1, 807 young people (aged 16–20 years, *M* = 17.1; 58% female, 33% male, 6% gender diverse, 3% did not say) completed a survey on social media use; recalled their exposure to alcohol, tobacco, and vape content and marketing within posts of influencers they follow; and named three favourite influencers. Among respondents who followed influencers (*n* = 665; 82.4%), 56% reported seeing influencer posts containing vapes (*n* = 347/620) and 23% vape marketing (*n* = 137/604); 47% reported seeing posts containing tobacco (*n* = 291/616) and 13% tobacco marketing (*n* = 78/604); and 76% reported seeing posts containing alcohol (*n* = 472/622) and 43% alcohol marketing (*n* = 260/602). Under-age respondents (16–17 years) reported seeing more tobacco marketing than older respondents (18–20 years). In Stage 2, researchers collected all Instagram posts of the 18 most frequently named influencers for 2 months and ephemeral ‘stories’ for 2 weeks. These posts/stories were analysed for harmful commodity content and marketing. Twelve influencers posted content with harmful commodities, primarily alcohol (117 posts, 6% of posts) but also vape and tobacco products (14 posts, 1% of posts). We need platform transparency and stronger policy to protect young people from harmful commodity marketing within influencers’ accounts.

Contribution to Health Promotion

- Digital marketing of harmful commodities influences consumption and is associated with negative health outcomes.
- Social media influencers are central to young people’s online worlds, but we know little about influencers’ promotion of harmful commodities.
- Young New Zealanders, including 16–17-year-olds, saw alcohol, vape, and tobacco content and marketing in the posts of influencers they follow.
- Popular influencers regularly posted alcohol (including their own alcohol brands) content, and less frequently vape and tobacco content, without any marketing disclosures.
- Social media require much stricter regulation to protect young people from the promotion and marketing of harmful commodities.

## Introduction

Industries that sell products with addictive or habituating properties use marketing to recruit young consumers (Courtwright 2019). Exposing children and young people to intensive marketing of health-demoting products (such as alcohol, vapes, and tobacco) in their digital worlds has long-term negative health consequences (Babor *et al.* 2022, Soraghan *et al.* 2023, Giesbrecht *et al.* 2024). Research on harmful commodity industries commonly includes tobacco, alcohol, food, and beverage and gambling industries (van den Akker *et al.* 2024). The commercial promotion of harmful commodities is subject to public oversight and control in most countries because these commodities have negative short-term impacts and long-term health effects on noncommunicable diseases (Babor *et al.* 2022, Soraghan *et al.* 2023, Giesbrecht *et al.* 2024). In the case of alcohol, the World Health Organization states that reducing marketing and promotion is one of the ‘best buys’ for reducing alcohol harm and that regulating cross-border marketing of alcohol—particularly through digital means—is particularly important (World Health Organisation 2024). Best buys also include comprehensive bans on tobacco advertising, promotion, and sponsorship, which have been successfully implemented globally through countries adopting the WHO Framework Convention on Tobacco Control (World Health Organisation 2024).

These global concerns are mirrored in Aotearoa New Zealand (NZ), where recent data show that alcohol, tobacco, and vapes remain key public health issues for young people. The 2024/2025 NZ Health Survey (*N* = 9253) found that 49% of 15–17-year-olds, and 79% of 18–24-year-olds, reported drinking alcohol in the previous year, while 8% of 15–17-year-olds, and 27% of 18–24 year olds, reported engaging in hazardous drinking (defined as an AUDIT score of 8 or higher; Ministry of Health 2025). While youth smoking rates have significantly declined over time, vaping has increased substantially with 35% of 15–17-year-olds, and 64% of 18–24-year-olds, having tried vaping in NZ. Further, 14% of 15–17-year-olds and 23% of 18–24-year-olds reported that they were vaping daily (Ministry of Health 2025).

Children and young people in NZ are also highly digitally connected. Research conducted in early March 2020 (just prior to a Covid-19 lockdown in NZ) showed that 82% of 6–14-year-olds in NZ use the internet each day. The most popular platforms used daily were YouTube, TikTok, Instagram, and Snapchat; 57% of 6–14-year-old children were using TikTok every day (NZ on Air 2020). In a 2022 survey with over 3600 young people in NZ (aged 14–20 years), 97% reported using the internet several times a day or almost constantly. Respondents also reported using between 1 and 19 different social media platforms in the past month, with an average of five platforms. The most commonly used platforms were Instagram, YouTube, Snapchat, and TikTok (Lyons *et al.* 2024b). Among Instagram users, 22% reported that they used the platform ‘almost constantly’, and 58% used it ‘several times a day’. These high levels of engagement demonstrate that social media platforms may be a key context in which young people are exposed to marketing content. Research shows that marketing on social media is highly effective with young people because it reaches them within their own online worlds (Lyons *et al.* 2017, 2023).

### Marketing harmful commodities to young people on social media platforms

Harmful commodity marketing is pervasive on social media platforms, reaching children and young people (Dunlop *et al.* 2016, Lyons *et al.* 2024, McCreanor *et al.* 2024). Alcohol marketing on social media that is targeted at young people is effective, impacting their attitudes, purchasing decisions, and consumption behaviours (Noel and Babor 2018, Rossen *et al.* 2018, Noel *et al.* 2020). Engaging with alcohol marketing on social media (by clicking on or sharing content, liking or following brands, etc.) has been positively related to young people’s frequency and amount of alcohol consumption (Noel *et al.* 2020, McCreanor *et al.* 2024). Similarly, vape product marketing has been identified on a range of social media platforms, has been seen by large numbers of children and young people (Hardie *et al.* 2023, Lyons *et al.* 2024), and has been associated with vaping behaviours, including purchasing vape products online (Lyons *et al.* 2024). Despite strict regulations around tobacco marketing by many governments, a 2017 study of Facebook found ‘widespread tobacco promotion and sales… at variance with the company’s policies governing advertising, commerce, page content and under age access’ (Jackler *et al.* 2019, p. 67).

The digital marketing efforts of large multinational alcohol and tobacco companies (transnational tobacco companies develop and sell a range of nicotine products, including vapes) have coevolved with the growing technical capacities of social media. Their experiments with branding and promotional content have continually pushed the boundaries of what can be achieved using the data-driven and algorithmic affordances of social media (Carah 2017, Carah and Brodmerkel 2021, Goodwin 2022). This has resulted in developments that blur the lines between user-generated and commercial content, making it difficult to identify digital marketing of harmful commodities as branded communications (Carah and Brodmerkel 2021). Branded social media pages provide powerful forms of promotions designed to work alongside competitions, games, branded filters, and user-generated content as covert strategies (Abidin 2016, Carter 2016, Gräve 2019). This marketing is often based in ephemeral content formats, which means it disappears from social media feeds rather than remaining publicly visible, and therefore is unavailable for public scrutiny and is not fully captured by marketing regulations or industry codes of practice (Carah and Brodmerkel 2021). These are some of the reasons that regulating marketing on online platforms is extremely challenging, as is enforcing marketing regulations.

### Influencer marketing

Recent years have seen a rise of influencer marketing on digital platforms. The global influencer marketing industry is vast, estimated to be worth US$480 billion by 2027 (Van Rossum 2024). Social media influencers are a substantial part of contemporary online cultures, sitting in-between ‘everyday’ users of social media and industry structures and practices (Brodmerkel and Carah 2013). Their online content can attract vast audiences, with some having millions of followers (Gräve 2019), although microinfluencers or microcelebrities also exercise influence through smaller, more dedicated followings (Marwick 2015, Abidin 2016).

Influencers target various groups, discuss specific topics, and importantly, succeed online through performing a carefully calibrated ‘authenticity’ through the ‘textual and visual narration of their personal lives and lifestyles’ (Abidin 2016, p. 1). This calibrated performance means that influencer culture, even when enacted online by celebrities with fame beyond social media, is marked by an ‘ordinariness’ in a way that departs from previous cultures of celebrity promotion. Influencers, even those with millions of followers, are often valued ‘as accessible, friendly, people next door who offer unbiased opinions and therefore possess high credibility and trustworthiness’ (Gräve 2019, p. 2).

Marketing campaigns use various algorithms and data sets to identify which influencers are appropriate for specific market segments. Metrics such as the nature and size of influencers’ audience reach, average ‘interactions’ stimulated by posts, and sentiment analysis are all part of these calculations (Gräve 2019). Sophisticated algorithms enable marketers to position products within target audiences’ everyday online social media feeds, making influencer marketing highly effective.

Influencers are powerful in broadcasting content, steering behaviour and shaping social norms (Ekinci *et al.* 2025). The relationship they cultivate with followers is built on trust, credibility, and authenticity (Dhingra *et al.* 2024), so their content is viewed as valuable, making their marketing more effective (Guégan *et al.* 2024, Ekinci *et al.* 2025). Additionally, because influencers post both commercial and noncommercial content, it can be difficult to distinguish marketing posts, which in turn makes it harder for followers to resist marketing messages compared to more traditional types of marketing (Jin *et al.* 2019). This is compounded by the fact that despite platform regulations, many influencers do not disclose marketing posts that are paid for or sponsored, nor do they comply with applicable laws (Musiyiwa and Jacobson 2023).

Multinational alcohol and tobacco corporations have used influencer marketing to target young people (Carah and Brodmerkel 2021, Nguyen Zarndt *et al*. 2023, Vassey *et al.* 2023, Lee *et al.* 2024). Research on influencers and alcohol has found that many popular influencers post about alcohol in ways where (branded) products and drinking are represented as positive, fun, and normal (Hendriks *et al.* 2020, Vranken *et al.* 2023, 2024, Guégan *et al.* 2024). A content analysis of Instagram posts of 178 popular Instagram influencers found that 63.5% had posted about alcohol recently, with alcohol featuring 3–4 times on average in their previous 100 posts (Hendriks *et al.* 2020). One-fifth of these posts clearly displayed an alcohol brand, but very few were disclosed as advertising. Further, a survey in the USA with undergraduate students found following influencers who posted alcohol-related content was associated with greater quantity and frequency of drinking (Strowger *et al.* 2024). This relationship was observed over and above exposure to peers posting alcohol-related content, and across different types of influencers, including actors, celebrities, and everyday people.

Influencers have also been employed to promote smoking to young people. For example, an Instagram influencer working for cigarette brand Lucky Strike shared aspects of their contract, including when they were required to have a cigarette in their posts, how many times they were required to post, the hashtags they had to use, and the nature of the smoking images that they had to promote (Green *et al.* 2019). Similarly, social media influencers have been employed to promote vaping and vape products to young people; Philip Morris International’s global campaign (illegally) used young influencers (under 25) to ‘sell its new IQOS [I Quit Ordinary Smoking] “heated tobacco” device’ (Dyer 2019). Despite platform policies that prohibit promoting vape products, such as Meta’s policy for Instagram, research has found that marketing of vapes by influencers is common (Silver *et al.* 2023, Vassey *et al.* 2023). The vape brand Vuse (owned by British American Tobacco) has been found to collaborate with influencers who have millions of followers to promote their brand (Hardie *et al.* 2025). A recent review on the role of social media influencers on adolescents’ health identified marketing of harmful products as a major challenge (Engel *et al.* 2024).

Governments have been developing policy and legislative responses to manage influencer marketing of harmful commodities. In NZ in 2021, the Advertising Standards Authority released guidance documents for influencers on how to correctly identify their advertising content on social media (Advertising Standards Authority 2021), adding additional disclosure requirements for influencers who also need to adhere to platform-specific policies. The code for influencers requires clear identification of advertisements and disclosure of payment or gifting within the first two lines of a caption. Additional restrictions are required for specific products to meet government regulations: alcohol marketing must not target minors or encourage excessive consumption. Vape marketing was prohibited in June 2025 but was permitted at the time of this study to anyone aged 18 or older. Tobacco marketing was and still is prohibited. In practice, we do not know how many advertisements are fully disclosed, and existing self-regulatory frameworks for harmful commodity marketing do not fully capture influencer marketing. It can be challenging to identify posts for which influencers are paid for promoting products and posts made as part of sharing their everyday lives that do not involve a commercial relationship requiring disclosure (Hendriks *et al.* 2020, Vranken *et al.* 2023).

### The current study

Currently, little is known about the broader influencer landscape, the influencers that young people follow, and whether these influencers post about and promote harmful commodities. The extent to which influencer marketing of alcohol, vape, and tobacco products reaches young people more broadly, including minors (those aged <18), in NZ is also unknown. This study addresses a critical gap by systematically examining young people’s recall of harmful commodity marketing within the posts of influencers they follow, identifying their favourite influencers and then independently examining alcohol, tobacco, and vape content and marketing within these accounts. The aims of the study were to:

1. Explore whether young New Zealanders—including those aged 16–17 years—recalled seeing alcohol, tobacco, and/or vape product marketing in influencer posts
2. Identify young people’s favourite influencers and document the most popular influencers among young people aged 16–20 years
3. Independently examine the social media posts of the most popular influencers for alcohol, tobacco, and vape content, as well as for alcohol, tobacco, and vape marketing, and the extent to which this marketing was disclosed, over a 2-month period.

## Materials and methods

### Stage 1: online survey with young people

An online Qualtrics survey was developed and piloted with young people to obtain information about 16–20-year-olds’ social media use, their favourite influencers, and their exposure to alcohol, cigarette, and vape content and product marketing. These products were selected because their harmful nature means that their marketing is either not permitted (in the case of cigarettes) or not permitted to target minors (those under 18 years) in NZ. Participation was voluntary and anonymous. The study received ethical approval from the Human Ethics Committee at Massey University, New Zealand.

#### Recruitment and procedure

As we were aiming for a diverse sample, we recruited potential respondents in 18 schools selected for their location (urban/rural), decile (low, middle, and high school rankings), and ethnic diversity in the lower North Island during 2021. We gave in-person presentations to classes/assemblies (two schools also shared a recorded video presentation). We also recruited in undergraduate classes in two universities and via recruitment posters. The survey took ∼15–20 minutes to complete. At the end of the survey, respondents could follow a link to anonymously enter a draw for one of three $100 gift vouchers.

#### Questionnaire

The questionnaire was constructed by adapting questions used in previous surveys with young people. This included demographic questions (adapted from the NZ Youth19 survey; Fleming *et al.* 2020), questions about internet and social media use [adapted from the Pew Research Center (Anderson and Jiang 2018) and the World Internet Project NZ study (Crothers *et al.* 2016)], and questions about exposure to harmful commodity marketing (Hoffman *et al.* 2014, Critchlow *et al.* 2019) and social media influencers (Lou and Kim 2019). The questionnaire was piloted using a ‘think aloud’ approach with 10 young people aged 16–18 years, adjusted for clarity, and finalized (available from the lead author on request). Respondents provided the following information:

1. Demographic—age, gender identity, ethnicity, living situation, geographic location (urban, rural, other), employment status, and whether they attend high school or university.
2. Internet connection and social media use—devices used, social media sites ever used, frequency of use, social media activities, favourite site, and disliked sites (and why).
3. Social media influencers—respondents were asked if they follow or see content from any influencers on social media. Influencers were defined in the following way:
  Influencers aren’t just on Instagram–they’re people who can be found on other social media including Facebook, YouTube, TikTok and so on. Across different platforms, influencers have some things in common. They tend to have built some form of reputation for their knowledge, opinions, interest, or expertise on a specific topic; regularly produce engaging content on their preferred social media channels; have followings of enthusiastic people who engage with their content; usually have large followings, from a few thousand to millions of people. We're also interested in who YOU find influential even if those people have fewer followers. Celebrities can also be influencers if you find them influential.
  Respondents were asked to name up to three of their favourite social media influencers. Subsequent questions asked about each named influencer, including the platform(s) they were followed on and the type of influencer they were (respondents selected all that applied from the following list: fashion, gaming, health, travel, lifestyle, food, pets, parenting, sports, activism, creative/art, music, other (please state), prefer not to say).
4. Exposure to vape/cigarette/alcohol product marketing—questions asked if the respondent recalled seeing any vape product advertising on each of the social media sites they had previously ever reported using; questions were repeated for cigarette and alcohol product advertising. Participants who followed social media influencers were asked whether they recalled ever seeing any posts that included alcohol, vape, or cigarette content, behaviours, or marketing within influencer posts.
5. Engagement with vape/cigarette/alcohol product marketing—if participants had seen vape product marketing, they were asked a series of questions about whether in the past month they had engaged with this marketing (liked a vape brand; shared something related to a vape brand; followed a vape brand; entered a competition run by a vape brand; searched for vape adverts). These questions were repeated for cigarette and alcohol product advertising.

#### Participants

The final sample consisted of 807 participants (58.1% female, 33.1% male, 6.1% as gender diverse, 2.7% preferred not to say) with an average age of 17.1 (SD = 1.14); 71% were aged 16–17 years, and 27.4% aged 18–20. Most (76.6%) were high school students, with 23.4% university students. There were a range of ethnicities, including Pākehā (NZ European 64.7%), Māori (19.8%), Pasifika (3.3%), and multiple ethnicities (21%). Most participants (69%) lived in an urban area, while 21.1% reported they lived rurally (10% did not say).

#### Analytic procedure

Data were screened for missing values and errors. Survey noncompleters (*n* = 121) were compared with completers (*N* = 807) on all demographic variables, and no significant differences were found; the final dataset included completers only. A series of descriptive analyses were undertaken in SPSS to describe participants’ social media use, exposure to and engagement with unhealthy product marketing, and marketing viewed on influencer accounts. To compare differences in reported exposure to vape, cigarette and alcohol marketing across age groups, age was dichotomized (16–17, 18–20), and a series of chi-square analyses were conducted. Statistical analyses were conducted using IBM SPSS Statistics v.20. Given the novelty and exploratory nature of the study, missing data were excluded (rather than imputed) from relevant analyses.

### Stage 2: analysis of influencer posts

We identified the 20 influencer accounts that were most frequently named in the sample. As Instagram was the most popular platform with respondents, we focused on these influencers’ Instagram accounts, monitoring and collecting their posts. Two of the 20 popular accounts were excluded from the analysis; one did not have an Instagram account at the time of the study (David Dobrik), while one was followed solely on YouTube (PewDiePie). We manually screen captured permanent image posts and related text in Instagram from the 18 influencer accounts over a 2-month period in 2022. The influencers posted many permanent and ephemeral videos, and our methods did not allow us to manually capture all of these. Therefore, we chose to monitor each account daily for a 2-week period and manually capture their Instagram ‘stories’ (where photographic and video content disappears after 24 hours) and not capture permanent videos. We used the Windows snipping tool to capture image posts, igram.io to capture video posts, and storysaver.net to capture Instagram stories.

We undertook a content analysis on posts to identify the presence of unhealthy products (alcohol, tobacco, vape). Where unhealthy products were included, we recorded product placement, format, and context in which product was shown (including people, ages, and activities), time of post (day, week, time of year), encouragement to engage with post, aesthetics, narrative, branding (clearly shown, mentioned), and advertising disclosure for each post. These codes were developed jointly by the multidisciplinary research team to capture relevant aspects of the posts, informed by their research expertise on influencers, marketing, and youth culture. To ensure reliability and consistency in coding, three members of the research team independently analysed five videos and coded them across 13 content areas. There were fewer than 10 discrepancies in coding across the 65 categories. Researchers discussed these discrepancies and developed more detailed definitions for some of the codes to ensure agreement. One researcher then undertook all the coding on the remaining posts; if they were unsure how to code any aspect, they checked with the other two team members. Posts/stories were defined as marketing if the brand of the product was clearly shown or mentioned.

## Results

### Stage 1: online survey

#### Internet and social media use

Survey respondents were high users of the internet, with almost all (98%; *n* = 791; seven respondents reported they did not use social media) indicating that they used the internet several times a day or almost constantly. They were also highly engaged users of social media platforms. The number of platforms ever used ranged from 0 to 14 (*M* = 7.4, median = 8). Social media that were most often visited by participants—at least once per day (including constantly, several times a day, and at least once per day)—were Instagram (87%; *n* = 690), Snapchat (74%; *n* = 589), YouTube (64%; *n* = 509), and TikTok (64%; *n* = 505). For more detailed information about participants’ internet and social media use specifically, please see the descriptive report associated with this dataset (Goodwin *et al*., 2024).

#### Social media influencers

Over four-fifths of respondents (83%; *n* = 665) reported that they followed an influencer or regularly saw their content and named up to three influencers that they follow. In total, 1571 influencer accounts were named, and after removing names that were generic (e.g. ‘musicians’) and those named more than once, 891 unique influencers were identified. This included 698 named once and 193 named two + (up to 45) times. Table 1 presents the 20 influencers who were named most frequently by respondents. These included established celebrities who actively use social media as well as those who gained celebrity through being influential on social media. As shown, the most frequently named influencers had large followings, with numbers ranging from millions through to a few hundred thousand followers. They came from six different countries (USA, UK, Australia, Ireland, Philippines, Sweden), demonstrating a diverse and international digital landscape.

**Table 1:** Top 20 influencer accounts identified by respondents.

Influencer	Respondents following			Influencer information		
	Total *N* = 807	16–17 yrs *n* = 573	18–20 yrs *n* = 221	Instagram follower count March 2022	Nationality	Category as most frequently categorized by respondents^a^
Emma Chamberlain	45	26	19	13 500 000	USA	Fashion
Kendall Jenner	29	23	6	181 000 000	USA	Fashion
Kylie Jenner	28	24	4	257 000 000	USA	Fashion
Addison Rae	18	14	4	39 700 000	USA	Fashion/lifestyle
Billie Eilish	17	14	3	90 000 000	USA	Music
Kim Kardashian	15	16	3	243 000 000	USA	Fashion/lifestyle
David Dobrik	14	11	3	12 900 000	USA	Lifestyle
Tana Mongeau	14	12	2	5 700 000	USA	Lifestyle
KSI (Olajide Williams)	13	11	3	10 400 000	UK	Gaming
PewDiePie (Felix Kjellberg)	12	9	3	21 600 000	Sweden	Gaming
The Sidemen (seven members, incl. KSI)	12	12	0	13 300 000	UK	Gaming
Bretman Rock (Bretman Sacayanan)	11	8	3	17 500 000	Philippines	Lifestyle
Markiplier (Mark Fischbach)	11	9	2	9 100 000	USA	Gaming
The Rock (Dwayne Johnson)	10	10	0	266 000 000	USA	Health^b^
Olivia Neill	10	7	3	732 000	Ireland	Lifestyle
Anna Paul	9	9	0	719 000	Australia	Lifestyle
Bella Hadid	9	8	1	44 800 000	USA	Fashion
Sarah’s Day (Sarah Stevenson)	9	5	4	1 200 000	Australia	Lifestyle
Zendaya (Zendaya Coleman)	9	6	3	103 000 000	USA	Fashion^b^
Harry Styles	8	6	2	39 000 000	UK	Music

^a^Respondents could choose more than one from the following list: fashion, gaming, health, travel, lifestyle, food, pets, parenting, sports, activism, creative art, music, other, and prefer not to say.

^b^There was no actor/acting response option.

Participants described the ‘type’ or ‘category’ of each influencer they named, and fashion and lifestyle influencers were most common, while gaming and music influencers were also popular [for further information about social media influencers followed, their content, and which demographic groups followed which influencers, see Goodwin *et al*. (2024)]. Many participants followed and engaged with influencers across more than one platform. Table 2 shows the platforms that respondents reported following their three named influencers on and how many respondents reported following influencers on each platform. Instagram, YouTube, and TikTok were the top platforms where influencers were followed and/or their content seen.

**Table 2:** Platforms where participants followed social media influencers (participants named up to three influencers each).

Platform	Number of responses	Percentage
Instagram	1226	36.4%
YouTube	842	25.0%
TikTok	516	15.3%
Twitter	206	6.1%
Snapchat	176	5.2%
Facebook	146	4.3%
Pinterest	89	2.6%
Reddit	39	1.2%
Facebook Messenger	20	0.6%
WhatsApp	14	0.4%
WeChat	8	0.2%
Other	82	2.5%
Total	3364^a^	–

^a^If each participant (*n* = 665) named only one social media platform for each of three influencers there would be no more than 1995 responses.

#### Unhealthy behaviours, products, and marketing in influencer posts

We asked respondents whether they saw vape, tobacco, and alcohol products, behaviour, and marketing within social media influencer posts. We prioritized respondents’ views on whether they saw marketing (rather than define it for them). Results are shown in Table 3. Respondents most commonly reported seeing alcohol products, drinking alcohol, and alcohol marketing (69%, 76%, and 43% of the sample who followed influencers, respectively, *N*s = 616, 622, 602) in influencer accounts, followed by vapes, vaping, and vape marketing (46%, 56%, and 23%, respectively, *N*s = 612, 620, 604). Tobacco products and smoking were also commonly seen by participants (33% and 47%, respectively, *N*s = 610, 616), although fewer (13%, *N* = 604) reported seeing tobacco marketing in influencer accounts. A series of chi-square analyses showed that there were no significant differences across the age groups (16–17 years compared to 18–20 years) in reporting exposure to alcohol and vape marketing, although younger respondents reported seeing more tobacco product marketing in influencer accounts than older respondents [chi-square analyses were also undertaken to check for demographic differences across the two age groups; they were not significantly different in terms of their gender (male, female, gender diverse), school decile (low, medium, and high), or ethnicity (NZ European, Māori, Pasifika, or another ethnicity)].

**Table 3:** Exposure to alcohol, vape, and tobacco products, behaviour, and marketing in social media influencer posts across age groups (16–17 years and 18–20 years).

Influencer posts that included	Respondents who had ever seen influencer posts that included unhealthy products								
	Full sample			16–17-year-olds			18–20-year-olds^a^		
	*N*	*N*	%	*N*	*n*	%	*N*	*n*	%
Vape products	612	283	46.2	443	209	47.2	159	69	43.4
Someone—or people—vaping	620	347	56.0	450	250	55.6	160	92	57.5
The marketing of vape products	604	137	22.7	436	104	23.9	158	28	17.7
Tobacco products	610	204	33.4	444	142	32.0	157	59	37.6
Someone—or people—smoking cigarettes	616	291	47.2	449	213	47.4	157	73	46.5
The marketing of tobacco products	604	78	12.9	439	65	14.8	155	10	6.5^a^
Alcohol products	616	426	69.2	443	312	70.4	163	109	66.9
Someone—or people—drinking alcohol	622	472	75.9	450	337	74.9	162	130	80.2
The marketing of alcohol	602	260	43.2	436	185	42.4	156	71	45.5

^a^A series of chi-square analyses showed that there were no significant differences across the age groups in reporting seeing these products and behaviours, except for tobacco product marketing, which was significantly more prevalent in younger respondents [chi-square (1, *N* = 594) = 7.25, *P* = 0.007, V = 0.11].

#### Stage 2: content analysis of Instagram posts/stories by the most frequently named influencers

Instagram posts of the 18 most popular influencers were analysed for harmful commodity content. Two of the top 20 were excluded due to not being on Instagram or followed on Instagram (David Dobrik did not have an Instagram account at the time of the study; PewDiePie was followed solely on YouTube). Six influencers did not include any alcohol, vape, or tobacco products in their posts. Table 4 provides an overview of the remaining 12 influencers and the number of posts they made that included harmful commodity marketing. In total, they posted 76 permanent image posts with alcohol content (6% of the 1355 total posts collected over the 2-month period) and 41 ephemeral ‘stories’ that included alcohol (6% of the 694 total ephemeral posts collected over the 2-week period). There were seven permanent image posts that included tobacco products (1% of total 1355 posts) and seven ephemeral ‘stories’ that included vape products (1% of the total 694 stories). There were posts that were explicitly and clearly showing alcohol, tobacco, and vape brands, but none of the influencers disclosed these posts as marketing. Unexpectedly, through this analysis, we identified four influencers who owned their own alcohol brand.

**Table 4:** Permanent and ephemeral Instagram posts that include alcohol, vape, and tobacco products by popular influencers.^a^

Influencer	Permanent image posts (2-month period)				Ephemeral image/video posts (2-week period)			
	All posts	Includes alcohol products	Includes vape products	Includes tobacco products	All posts	Includes alcohol products	Includes vape products	Includes tobacco products
Emma Chamberlain	50	7	0	0	10	0	0	0
Kendall Jenner^b^	60	4	0	0	41	5	0	0
Kylie Jenner	129	8	0	0	97	0	0	0
Addison Rae	129	1	0	0	129	0	0	0
Kim Kardashian	235	5	0	0	92	1	0	0
Tana Mongeau^b^	72	19	0	0	123	23	7	0
KSI^b^	16	1	0	0	12	1	0	0
Sidemen	85	11	0	1	10	2	0	0
Bretman Rock	148	0	0	3	49	0	0	0
Dwayne Johnson^b^	89	16	0	0	6	3	0	0
Olivia Neill	10	1	0	0	47	5	0	0
Bella Hadid	332	3	0	3	78	1	0	0
Total	1355	76 (6%)	0	7 (1%)	694	41 (6%)	7 (1%)	0

^a^Six influencers did not post any alcohol, vape, or tobacco content (Billie Eilish, Markiplier, Anna Paul, Sarah’s Day, Zendaya, and Harry Styles), while two influencers were excluded from this analysis: David Dobrik did not have an Instagram account at the time of the study; PewDiePie was followed solely on YouTube.

^b^Had own alcohol brand at the time of study.

## Discussion

This study provides insight into young people’s digital worlds, particularly the social media influencers they follow and the extent of harmful commodity content and marketing they see within influencer accounts. It is the first study of its kind to identify the popular influencers followed by young New Zealanders and the first to analyse influencer content in this way. Respondents most commonly reported seeing alcohol content and marketing in influencer accounts, followed by vape and tobacco content and marketing. This content was recalled by many who are under-age and who legally should not have these products marketed to them. Crucially, it demonstrates that influencer content related to vape, alcohol, and tobacco products evades current regulatory regimes. Young respondents reported seeing more tobacco product marketing than older respondents when neither should be receiving any marketing of these products under the New Zealand Smokefree Environments and Regulated Products Act 1990 (New Zealand Government 1990). Tobacco companies are known to target younger audiences to recruit future generations of users (WHO 2024). Findings also highlighted that influencers who are popular with young people post alcohol marketing and to a lesser extent vape and tobacco marketing (where the post is promotional with the brand explicitly shown) without any marketing disclosure.

Young people in this study regularly used a large number of social media platforms and followed a diverse set of (mostly international) influencers. Social media influencers use multiple platforms and tailor their content to suit the platform within a broader social media ecology (Goodwin and Lyons 2019). For example, influencers posting vape use and promotion have been found to be more frequently followed by teenagers on TikTok and Pinterest and by young adults on Twitter/X (Lee *et al.* 2024). In the current study, social media influencers who are popular with young people posted images that included alcohol and drinking, consistent with previous research on alcohol (Guégan *et al.* 2024, Strowger *et al.* 2024), and to a lesser extent tobacco and vapes (Vassey *et al.* 2020, 2023, Lee *et al.* 2024). We also found lifestyle influencers frequently posted about alcohol, similar to previous research (Hendriks *et al.* 2020). This content normalizes these products as part of young people’s social worlds, where they spend significant amounts of time and where they follow their favourite influencers. Influencers have been found to have high credibility and trust with adolescent followers, and influencers’ behaviours and endorsements have been shown to impact the attitudes and behaviours of their followers (Engel *et al.* 2024, Powell and Pring 2024).

Relatively little research has examined social media influencers and marketing of unhealthy products, possibly because many are bound by contractual agreements that do not allow disclosure of this activity, and they post images and videos that are often ephemeral (e.g. Instagram reels disappear after 24 hours). We found that 6% of Instagram stories posted by 12 popular influencers who posted harmful commodity content included alcohol and drinking. Further, four of the 20 most popular influencers named by participants had their own alcohol brand that they promoted online without the commercial disclosures required by third-party advertising. This makes it difficult to distinguish between noncommercial and marketing content and is especially concerning as their efforts to craft a personal brand that is attractive to young people become conflated with the alcohol brand they own and promote. This requires further study given the large numbers of followers that these popular influencers have and the fact that followers include those aged under 18 years who should not be receiving alcohol marketing within their social media feeds.

The current study has a number of limitations. We intentionally and successfully recruited a relatively diverse sample, although it was a convenience sample of students at high schools and universities in the lower part of the North Island, so findings cannot be generalized to other young people in Aotearoa or more globally. The study was cross-sectional, so temporal associations cannot be shown. It also relied on young people’s self-reports of whether or not they recalled seeing the marketing of unhealthy products within influencers’ posts. This recall could have been influenced by time spent online, recalling only recent exposure, or being particularly attentive to particular products (e.g. because of age). Further, within the survey, respondents were asked about influencers they follow before answering questions regarding alcohol and vape marketing, which may have led to over-reporting of seeing marketing on influencer accounts (misattributing the source of their memories of marketing to influencer accounts because they had already named influencers). Some marketing also may have been outside awareness, ambiguous, not noticed, ignored, or not remembered. The validity of self-report measures when compared to objective log measures has been questioned (Parry *et al.* 2021), including when respondents report seeing alcohol content (Riordan *et al.* 2023). However, we are most interested in what young people do recall seeing within their own digital environments and what has an impact on them. New data collection methods are emerging that include automatic detection of digital advertisements of harmful commodities and users taking and sharing screen shots of such marketing (Angus *et al.* 2024, Rutherford *et al.* 2024, McGlinchy *et al.* 2025). Future research would benefit from asking young people to share marketing content they view in their social media feeds in real time and taking a holistic approach across social media given that young people regularly use multiple platforms and are likely to see different content on each, as well as follow different influencers on different platforms.

Social media platforms operate in a way that mean that researchers cannot access the content that appears within users’ social media feeds, so marketing is occluded from view from researchers (unless people share it with them) (Goodwin 2022). Generally, there is a lack of transparency of commercial content on social media platforms, and most platforms do not allow researchers to fully access such content nor to determine if all marketing is properly disclosed (Lyons *et al.* 2023). Each platform also has its own policies around regulating content, including influencer content, although these are not consistently enforced (Lee *et al.* 2024); our research provides further evidence of this ineffectual regulation.

The marketing of alcohol and vape products has been linked to young people’s use of these products, including starting to use them at younger ages and how much and how often they use them (and for alcohol, problematic use later in life), which in turn impacts on the development of noncommunicable diseases (Babor *et al.* 2022, Soraghan *et al.* 2023, Giesbrecht *et al.* 2024). Therefore, we need more rigorous policies and greater enforcement by social media companies to limit young people’s exposure to unhealthy product marketing. This is particularly the case for regulating influencer marketing, which remains challenging globally as their content circulates across national borders, as evidenced by the current study. Importantly, young people aged 16–17 years reported seeing alcohol, tobacco, and vape marketing, even though there are regulations against marketing these products to those aged under 18 in NZ. Concerningly, they also reported seeing significantly more tobacco marketing within influencer accounts than those aged 18–20 years. The UN Convention on the Rights of the Child and the UN General Comment in relation to Digital Environments (General Comment 25) call on governments to take a rights-based approach to regulating the online environment, requiring governments to respect, protect, and fulfil the rights of all children in digital environments (UN 1989, UN Committee on the Rights of the Child 2021). This includes protecting children from exposure to harmful marketing, such as tobacco and alcohol.

The concept of limbic platform capitalism theorizes how social media platforms use a limbic model to capture and sustain users’ attention by stimulating the brain’s circuits related to mood, pleasure, habit, and addiction (Lyons *et al.* 2023). Platforms target users based on their emotions and desires (such as pleasure and escape) and ensure that flows of information keep users engaged. Key features of social media platforms, such as personalized algorithmically driven feeds, stimulate the limbic system of the brain in order to maximize engagement—which also maximizes exposure to marketing content. The pleasurable affect makes it increasingly difficult for people to moderate their use of these platforms (Courtwright 2019). Keeping users online produces surplus data that can be then sold to marketing companies (Zuboff 2019). Thus, social media platforms are designed to keep users online and encourage them to engage with promotional content and share it across their networks. This includes engaging with the marketing and promotion of addictive products, such as alcohol, tobacco, and vapes (Lyons *et al.* 2023).

Considered within limbic platform capitalism, the current findings illustrate the intersection of the commercial and digital determinants of health (Pitt *et al.* 2024). Policy urgently needs to be redesigned to take social media, influencers, and other aspects of the digital world into account to protect people from harmful commodities (Sing and Lyons 2024). This might take the form of a ban on harmful commodity marketing of alcohol, vapes, and tobacco on social media platforms. Banning such marketing to minors specifically is legislated in many countries, but the global nature of platforms and influencers means that such content frequently falls outside local jurisdictions. A full ban on specific products is possible. For example, a full ban on all paid-for digital advertising of high fat, salt, sugar foods has been legislated in the UK and is due to come into force in January 2026. This may prove a valuable leading example of how such bans can be adopted and implemented (Department of Health and Social Care UK 2025).

The findings of this study demonstrate that our current regulation is not effective in protecting our young people from the harms of unhealthy product marketing on social media. We echo others who call for multisectoral action and policy responses from governments, as well as social media and other Big Tech companies. Platforms should be required to be fully transparent regarding marketing exposure metrics and remove content that is in breach of the law. We also call for tighter regulation of influencer marketing to protect minors and for penalties to be enforced when influencers fail to disclose promotion of harmful commodities. Liability could be extended to all of those involved in the digital marketing supply chain. Multiple approaches are required to address structural inequities and oppose the health-harming industries that promote alcohol, tobacco, and vape to children and young people via social media influencers.

## Conclusion

This study advances our understanding of the influencers that are popular with young New Zealanders and the extent to which these influencers (with vast numbers of followers) post content promoting alcohol, tobacco, and vape products. It highlights that young people, including minors, recall being exposed to alcohol, tobacco, and vape marketing through globally popular influencer accounts and that these posts were never disclosed as marketing among the subset of 18 most popular influencers whose content was studied. In NZ, those aged younger than 18 years should not be exposed to such marketing, demonstrating current regulations are ineffective. Multisectoral policy responses could include a full ban on the digital marketing of harmful, addictive commodities and stronger monitoring and enforcement of penalties for social media influencers who disregard marketing regulations.

## Data Availability

The data used in this article cannot be shared publicly because participants consented to only the research team having access to their survey responses.
